# Diagnostic Efficacy and Tolerability of Molded Plastic Nasopharyngeal Swab (FinSwab) Compared to Flocked Nylon Swab in Detection of SARS-CoV-2 and Other Respiratory Viruses

**DOI:** 10.1128/Spectrum.00736-21

**Published:** 2021-10-20

**Authors:** Miia Laine, Jaakko Ahti, Ville Peltola, Piritta Peri, Antti J. Hakanen, Matti Waris

**Affiliations:** a Department of Clinical Microbiology, Turku University Hospital, Turku, Finland; b Institute of Biomedicine, University of Turku, Turku, Finland; c Department of Paediatrics and Adolescent Medicine, University of Turku, Turku, Finland; d Department of Paediatrics and Adolescent Medicine, Turku University Hospital, Turku, Finland; Johns Hopkins Hospital

**Keywords:** COVID-19, nasopharyngeal swab, plastic injection molding, respiratory viruses

## Abstract

The supply of testing equipment is vital in controlling the spread of SARS-CoV-2. We compared the diagnostic efficacy and tolerability of molded plastic (FinSwab; Valukumpu, Finland) versus flocked nylon (FLOQSwab; Copan, Italy) nasopharyngeal swabs in a clinical setting. Adults (*n* = 112) with suspected symptomatic COVID-19 infection underwent nasopharyngeal sampling with FinSwab and FLOQSwab from the same nostril at a drive-in coronavirus testing station. In a subset of 36 patients the samples were collected in a randomized order to evaluate the discomfort associated with sampling. SARS-CoV-2 and 16 other respiratory viruses, as well as human β-actin mRNA were analyzed by using reverse transcriptase PCR (RT-PCR) assays. Among the 112 patients (mean age, 38 [standard deviation (SD), 14] years) β-actin mRNA was found in all samples. There was no difference in the β-actin mRNA cycle threshold (*C_T_*) values between FinSwab (mean, 22.3; SD, 3.61) and FLOQSwab (mean, 22.1; SD, 3.50; *P* = 0.46) swabs. There were 31 virus-positive cases (26 rhinovirus, 4 SARS-CoV-2, and 1 coronavirus-OC43), 24 of which were positive in both swabs; 3 rhinovirus positives were only found in the FinSwab, and similarly 4 rhinovirus positives were only found in the FLOQSwab. Rhinovirus *C_T_* values were similar between swab types. Of the 36 patients, 22 (61%) tolerated the sampling with the FinSwab better than with the FLOQSwab (*P* = 0.065). The molded plastic nasopharyngeal swab (FinSwab) was comparable to the standard flocked swab in terms of efficacy for respiratory virus detection and tolerability of sampling.

**IMPORTANCE** We demonstrate that a molded plastic swab is a valid alternative to conventional brush-like swabs in collection of a nasopharyngeal sample for virus diagnostics.

## INTRODUCTION

As a response to the coronavirus disease 2019 (COVID-19) pandemic, unprecedented actions were taken throughout the world to limit the transmission of the causative agent, severe acute respiratory syndrome coronavirus 2 (SARS-CoV-2) (1–4). Among these actions, extensive diagnostic testing has been a key element. The gold standard for diagnostic testing of SARS-CoV-2 is the reverse transcriptase PCR (RT-PCR) test on a specimen collected from the nasopharynx with a nasopharyngeal swab (5). The need for testing expanded exponentially in the early months of the pandemic, leading to a worldwide shortage of equipment, including nasopharyngeal swabs, which were particularly vulnerable since the global supply depended on only a few suppliers (6–8). Consequently, the lack of nasopharyngeal swabs caused a bottleneck into the supply chain for the diagnostics, greatly damaging the SARS-CoV-2 testing capability (9).

To address this matter, we collaborated with the Finnish company Valukumpu Oy in designing a new type of nasopharyngeal swab. The first prototypes were manufactured using three-dimensional (3D) printing technology, which has aroused wide interest as a possibility for nasopharyngeal swab manufacturing during the pandemic (6–11). The final product, model VK7 (FinSwab), was produced by plastic injection molding, which is better suited for the mass production of swabs than 3D printing.

We evaluated the efficacy of the FinSwab nasopharyngeal swab for the diagnosis of SARS-CoV-2 and other respiratory viruses in a clinical setting at a drive-in COVID-19 testing station using flocked nylon nasopharyngeal swabs (FLOQSwab; Copan, Italy) as the reference swab. We also assessed the discomfort of sample collection by FinSwab compared to FLOQSwab in a randomized substudy. Our study demonstrates the efficacy and convenience of FinSwab nasopharyngeal swab.

## RESULTS

A total of 115 subjects fulfilling the inclusion criteria were enrolled. After collection of the first swab specimen, three patients declined collection of the second specimen. Thus, 112 patients were included in the study (Table 1). The mean age of participants was 38 (standard deviation [SD], 14) years (range, 18 to 76), and 67% were female. The clinical setting was a drive-in testing station adhering to the national guidance to test all individuals with respiratory or other symptoms suggesting a possibility of COVID-19; in this setting the patients were symptomatic but ambulatory, and most of them had relatively mild symptoms.

**TABLE 1:** Demographic and diagnostic characteristics of the 112 study subjects

Characteristic	*n* (%)^*a*^
Participants	
First part of the study	76 (68)
Second part of the study	36 (32)
Gender	
Female	75 (67)
Male	37 (33)
Mean age, yr (range)	38 (18–76)
Virological result	
Positive^*b*^	30 (27)
Rhinovirus	26 (23)
SARS-CoV-2	4 (4)
CoV-OC43	1 (1)
Negative	82 (73)

a Unless otherwise stated.

b For at least one virus. One participant was positive for rhinovirus and CoV-OC43.

**FIG 1:**
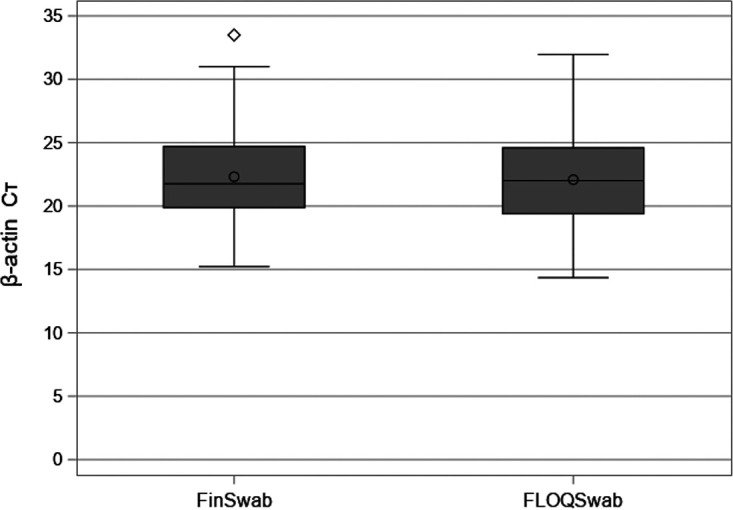
Human β-actin mRNA *C_T_* values from FinSwab and FLOQSwab in 112 patients. Medians, interquartile ranges, and minimum and maximum values are presented. The means are marked with a dot. An outlier is marked with diamond.

Human β-actin mRNA was found in all specimens collected either with FinSwab or FLOQSwab. The β-actin mRNA cycle threshold (*C_T_*) data were normally distributed. There was no difference in β-actin mRNA *C_T_* values between FinSwab (mean, 22.3; SD, 3.61) and FLOQSwab (mean, 22.1; SD, 3.50) specimens (*P* = 0.46; Fig. 1).

**FIG 2:**
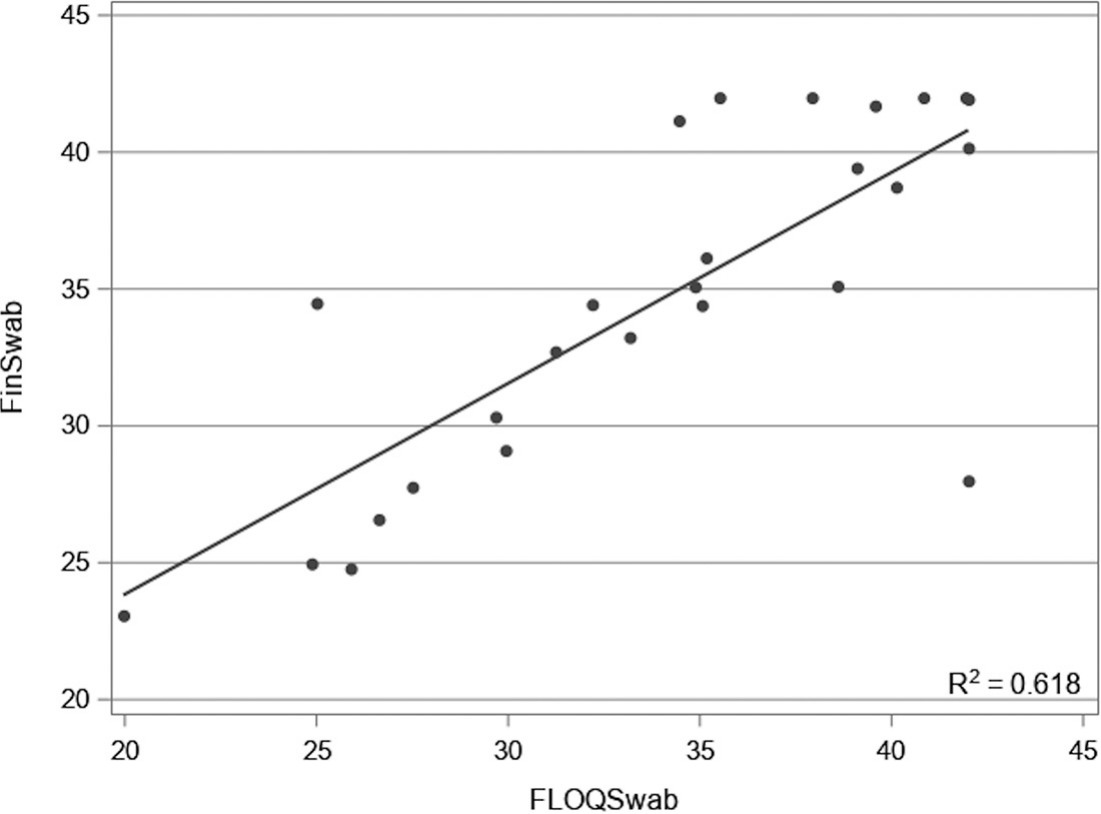
Correlation between rhinovirus RNA *C_T_* values from FLOQSwab and FinSwab.

Rhinovirus was detected in 26 patients. Of these, 19 cases were positive with both swabs. Three cases were positive only with the FinSwab, and four cases were positive only with the FLOQSwab. Of the seven discrepant results, five were weakly positive. Rhinovirus RNA *C_T_* values of cases detected only with FinSwab were 28.00, 40.16, and 41.94. *C_T_* values of cases detected only with FLOQSwab were 35.51, 41.93, 37.92, and 40.83. The rhinovirus RNA *C_T_* data were not normally distributed; they was skewed toward higher values. There was no difference in rhinovirus RNA *C_T_* values between FinSwab (median, 34.8; interquartile range [IQR], 28.6 to 41.0) and FLOQSwab (median, 35.0; IQR, 29.7 to 40.3; *P* = 0.12). A strong positive correlation was observed in the rhinovirus RNA *C_T_* values between the swabs (*r* = 0.786; Fig. 2).

Of the four cases of SARS-CoV-2, all were detected with both swabs. The mean SARS-CoV-2 RNA *C_T_* values for FinSwab and FLOQSwab were 19.5 (SD, 3.90) and 17.7 (SD, 3.96), respectively. Coronavirus OC43 was detected in one patient with both swabs (*C_T_* values, 24.6 and 26.2 for FinSwab and FLOQSwab, respectively).

In the first part of the study, the swabs were transported using dry tubes or tubes with viral transport medium (VTM). When analyzing the swabs transported in dry tubes, there was no difference in β-actin mRNA *C_T_* values between FinSwab and FLOQSwab (Table 2). In swabs transported in VTM, β-actin mRNA *C_T_* values of FinSwab (mean, 22.0; SD, 2.35) were slightly lower than those of FLOQSwab (mean, 23.0; SD, 2.60), but the difference was not statistically significant (*P* = 0.080; Table 2).

**TABLE 2:** Human β-actin mRNA *C_T_* values in FinSwab and FLOQSwab from the first part of the study^*a*^

Transport tube	FinSwab, mean *C_T_* (SD)	FLOQSwab, mean *C_T_* (SD)	*P* value
Dry (*n* = 48)	20.5 (2.93)	20.0 (2.99)	0.306
Viral transport medium (*n* = 28)	22.0 (2.35)	23.0 (2.60)	0.080
Total (*n* = 76)	21.0 (2.81)	21.1 (3.20)	0.860

a Swabs in the dry tubes were suspended in 1 ml PBS, whereas the volume of viral transport medium was 3 ml. FLOQSwabs were collected first.

**FIG 3:**
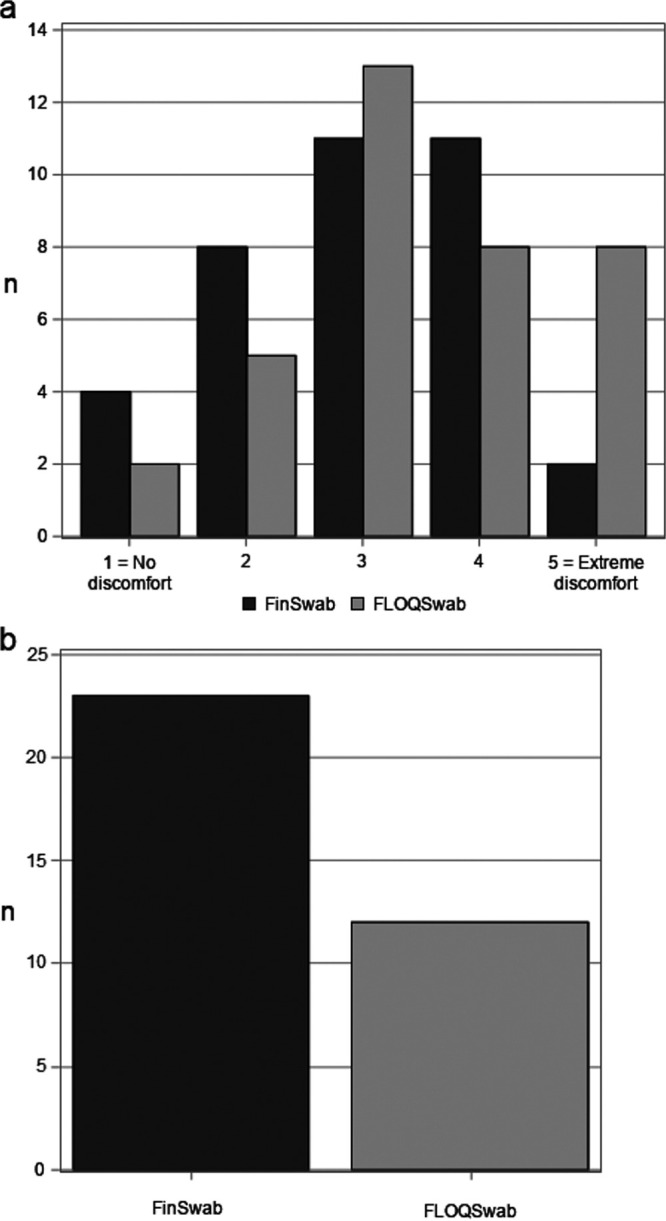
(a) The discomfort associated with nasopharyngeal sampling by FinSwab and FLOQSwab, as assessed by the patients with a discomfort scale from 1 (no discomfort) to 5 (extreme discomfort). (b) Preferred nasopharyngeal swab by the patients.

In the second part of the study, sampling discomfort was evaluated in 36 patients. On the discomfort scale, indicating better tolerability with lower values, 22 patients gave lower discomfort value for FinSwab, 10 did so for FLOQSwab, and 4 patients reported equal values for both (*P* = 0.065; Fig. 3a). The median values on the scale (FinSwab, 3.0 [IQR, 2.0 to 3.0]; FLOQSwab, 3.0 [IQR, 3.0 to 4.0]) were similar.

When asked which of the swabs the patient would prefer if a further nasopharyngeal sampling was needed, 23 (64%) of the 36 patients selected FinSwab, 12 (33%) selected FLOQSwab, and 1 patient could not decide between the swabs (*P* = 0.063; Fig. 3b).

No adverse events concerning the sampling with FinSwab or FLOQSwab were reported in any part of the study.

## DISCUSSION

The COVID-19 pandemic has overstretched the public health care system to its limits in many countries (12), forcing researchers and health care professionals to find novel solutions for acute material shortages. The first wave of the pandemic in spring 2020 caused a shortage of nasopharyngeal swabs around the world. Our comparative study demonstrates FinSwab as a valid alternative to the standard flocked nylon nasopharyngeal swab and shows that a plastic injection molding technique is suitable for production of nasopharyngeal swabs for diagnostic purposes. We found that FinSwab equals the diagnostic efficacy of the widely used reference swab, Copan FLOQSwab, in detecting human β-actin mRNA, rhinovirus RNA, and SARS-CoV-2 RNA. We also found that FinSwab can be transported in a dry transport tube or in VTM without substantially affecting its performance. It is important to note that FinSwab and FLOQSwab were similarly tolerated by the patients, as assessed by the discomfort scale and swab preference.

The prototypes of FinSwab were 3D printed. There are several advantages to 3D printing, including the ability to produce swabs swiftly, which was an essential feature for initiating the process without further delays (13). Several studies have found 3D-printed swabs to be equal to standard swabs for detection of SARS-CoV-2 (7–11), emphasizing the applicability of 3D-printing technology in the medical setting. Compared to 3D printing, plastic injection molding demands more initial time and investment but has several potential advantages—suitability to high-volume production of small objects, repeatability, scalability, and versatility of the process (14, 15). To our knowledge, the plastic injection molding technique has not been previously used in the production of nasopharyngeal swabs.

We used human β-actin mRNA and rhinovirus *C_T_* values as our main outcome, while most other studies of novel swabs have focused only on SARS-CoV-2 (7–11). In one study, a novel 3D-printed swab was compared to a control swab in laboratory conditions using respiratory syncytial virus in VTM (8). Human β-actin mRNA *C_T_* values were equivalent between the swabs, which supports our hypothesis that the FinSwab and the control swab collect equal amounts of cellular material from human nostrils. Rhinovirus was detected with similar sensitivity, and *C_T_* values were similar in specimens collected with both swabs, which is in line with the analysis of SARS-CoV-2 in other studies evaluating 3D-printed swabs (7, 9). Similar to SARS-CoV-2, rhinovirus is a single-stranded positive-sense RNA virus, which suggests that FinSwabs should be compatible with SARS-CoV-2 diagnostics as well.

Discomfort caused by nasopharyngeal sampling is an important but often overlooked aspect to evaluate, as recently pointed out by Locher et al. (16). In our study, most patients found nasopharyngeal testing at least mildly unpleasant regardless of the swab type. Substantial discomfort could lead to patients not being willing to be tested when necessary. In an Australian study, Williams et al. (11) evaluated the discomfort of sampling in 50 health care workers in an unblinded setting, and found 3D-printed swabs to be less uncomfortable than standard swabs. In our randomized assessment of discomfort, we obtained comparable results between FinSwab and FLOQSwab. A higher percentage of subjects preferred FinSwab over the control swab, but this finding was statistically insignificant.

There are strengths in our study. In addition to SARS-CoV-2, we tested for rhinovirus and other respiratory viruses. By analyzing human β-actin mRNA, we demonstrated in the first part of the study that there was no bias due to swabbing with the flocked swab first. Performing the study in a real-world clinical setting at a drive-in testing station with the nasopharyngeal swab specimens being collected by the attending nurses increases the generalizability of our results to other corresponding settings. Our randomized study design for the tolerability of the sampling reduced the bias.

There are also several limitations in our study. First, there were only 4 SARS-CoV-2-positive patients in our study, which was due to there being only moderate epidemic activity of COVID-19 and a low threshold for sampling in Finland during the study period. Even though rhinoviruses were frequently found in our study, the sample size was quite small. There was some discrepancy between the swabs in the rhinovirus detection, involving particularly poorly repeatable weakly positive results. Second, the evaluation of discomfort could have been affected by selection bias since people who find nasopharyngeal sampling very unpleasant were probably more likely to refuse to participate in a study like this with two swabs, making the study population skewed. Third, the discomfort evaluation could not be made double-blinded because sampling requires a visual control of the swab, and for practical reasons, the subjects only had their eyes closed, instead of being blindfolded.

To conclude, in this study the molded plastic nasopharyngeal swab, FinSwab, was found to be comparable to the standard flocked swab regarding both the swab’s diagnostic efficacy for respiratory virus detection and tolerability of sampling.

## MATERIALS AND METHODS

### Swab design.

**FIG 4:**
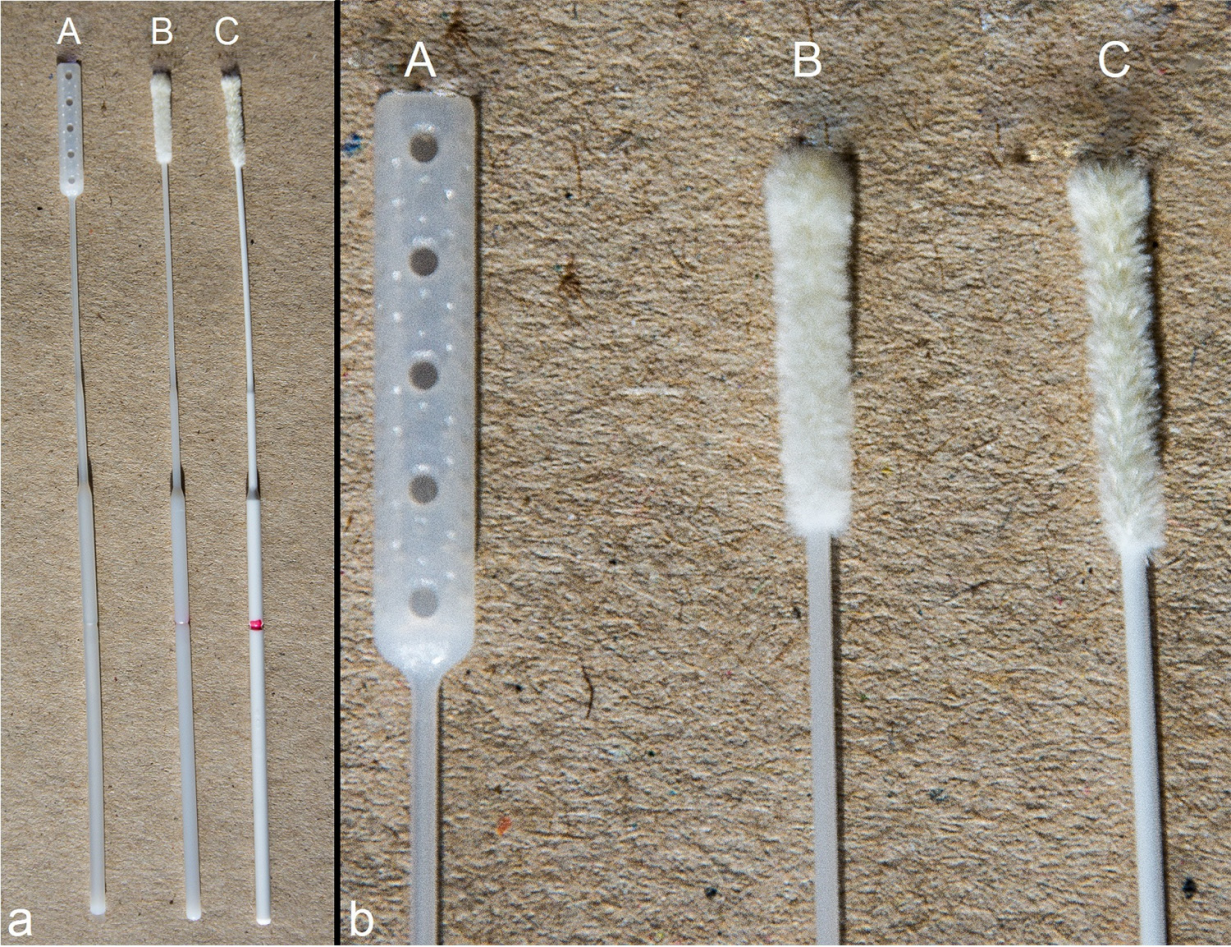
Nasopharyngeal swabs used in the study. (A) FinSwab; (B) Copan FLOQSwab 503CS01; (C) Copan FLOQSwab XA5S108B01. FinSwab was manufactured using a plastic injection molding technique by Valukumpu Oy, Finland.

Nasopharyngeal swab prototypes and final products were manufactured by Valukumpu Oy (Finland). The initial prototypes were 3D-printed swabs from which the final nasopharyngeal swab (model VK7, FinSwab; Fig. 4 and Fig. S1 in the supplemental material) was chosen based on *in vitro* testing and a pilot study measuring functionality and comfort of use in healthy adult volunteers (*n* = 10). The final product was manufactured using a plastic injection molding technique.

### Study population and conduct.

We recruited ambulatory patients at a COVID-19 drive-in testing station of the Turku University Hospital, Turku, Finland, in October 2020 and February 2021 when the local COVID-19 epidemic activity was moderate. The inclusion criteria were (i) age ≥18 years and (ii) need for a SARS-CoV-2 PCR test due to any symptom suggesting COVID-19. There were no exclusion criteria. Swab specimens were collected from the nasopharynx according to the instructions of the Turku University Hospital by the attending nurses. In the first part of the study, the nasopharyngeal specimen was first taken with the reference swab (Copan flocked nylon swab [FLOQSwab] model 503CS01, or model XA5S108B01 in the Cepheid Xpert SWAB/B-100 specimen collection kit) and then with FinSwab from the same nostril to prevent a possible bias caused by varying anatomy of the nostrils. The effect of the transport medium on the diagnostic efficacy was compared by collecting the swabs in dry transport tubes or in tubes with Xpert viral transport medium (VTM; Cepheid, USA) (Table 3).

**TABLE 3:** Nasopharyngeal swabs and transport tubes used in the study

Study part	Swab(s)	Transport tube	No. of paired samples
1	FinSwab FLOQSwab 503CS01	Sarstedt 60.540.016 (dry tube)	48
1	FinSwab FLOQSwab XA5S108B01	Xpert viral transport medium SWAB/B-100	28
2	FinSwab FLOQSwab 503CS01	Sarstedt 60.540.016 (dry tube)	36

In the second part of the study, we evaluated the discomfort associated with nasopharyngeal sampling at the same drive-in testing station. The reference swab (Copan FLOQSwab model 503CS01) and FinSwab were collected in a randomized order from the same nostril. In detail, after giving a written consent, subjects were led to a specified testing line. First, a nurse performing the upcoming testing explained it to the subjects and then asked the subjects to close their eyes. After that, an assisting nurse opened the swab packages and gave nasopharyngeal swabs to the performing nurse one by one. The performing nurse observed the subjects during the process, so that they would not peek at any time. After both specimens were collected and put into the transport tubes, subjects were allowed to open their eyes. They were then asked to assess the discomfort caused by the swabbing on a scale from 1 (no discomfort) to 5 (extreme discomfort). After this procedure, the study nurse asked the patients which of the swabs they would prefer if further nasopharyngeal sampling was needed.

All the study participants provided written informed consent before enrollment. The study protocol was approved by the Ethical Committee of the Hospital District of Southwest Finland (no. 21/1801/2020).

### Diagnostic methods.

Swabs in dry tubes were suspended in 1 ml of phosphate-buffered saline (PBS), vortexed, and settled for 10 min. An aliquot of 300 μl of the specimen in PBS or VTM was subjected to nucleic acid extraction performed with a PerkinElmer Chemagic viral DNA/RNA 300 kit H96 using a PerkinElmer Chemagic 360 extractor (PerkinElmer Wallac, Finland) with an elution volume of 70 μl.

A SARS-CoV-2 quantitative RT-PCR (qRT-PCR) was performed using WHO-recommended primers and probe for E gene (17) with Bioline SensiFAST probe one-step master mix (Meridian Bioscience, USA) in a Mic qPCR cycler (Bio Molecular Systems, Australia). For each 25-μl reaction, 5 μl of nucleic acid was used. The cycle threshold (*C_T_*) results were interpreted as follows: *C_T_* < 38, positive; *C_T_* 38 to 40, equivocal; *C_T_* > 40 negative.

After the SARS-CoV-2 PCR, the nucleic acids were stored at −80°C for further analyses. A multiplex RT-PCR assay (Allplex respiratory panels 1 to 3; Seegene, Republic of Korea) was performed according to the manufacturer’s instructions in a Bio-Rad CFX96 instrument to detect 16 respiratory viruses—rhinovirus, adenovirus, enteroviruses, coronaviruses 229E, NL63, and OC43, human metapneumovirus, human bocavirus, influenza A and B viruses, parainfluenza virus types 1, 2, 3, and 4, and respiratory syncytial viruses A and B. *C_T_* results were interpreted as follows: *C_T_* < 37, positive; *C_T_* 37 to 42, weakly positive; *C_T_* > 42 negative.

To measure human cellular material in the nasopharyngeal samples, a qRT-PCR for human β-actin mRNA was performed with slight modifications to the previously published protocol (18); Bioline SensiFAST probe one-step master mix (Meridian Bioscience, USA) was used in the protocol, and analyses were performed with a Rotor Gene 3000 (Qiagen, Germany).

### Statistical analysis.

The statistical analyses were performed using SPSS Statistics 26 (IBM, USA). Percentages were compared with the χ^2^ test, means with the paired or independent two-sample *t* test as applicable, and medians with the Mann-Whitney U test or with the Wilcoxon signed rank test. Pearson’s correlation coefficient was used to correlate *C_T_* values in FLOQSwab and FinSwab specimens from each patient. When analyzing quantitative PCR results for rhinovirus, a *C_T_* value of 42.0 was given for negative results in cases where rhinovirus was found with only one swab. Evaluation of the discomfort associated with the sampling was performed by collection of two swabs from each participant in a random order. The randomization code was generated using SAS 9.4 for Windows (SAS Institute, USA) using a random permuted block randomization, resulting in a collection of swab types where both swabs had equal times as the first and the second nasopharyngeal swab.
