# Supplementary figures and images for: Diagnostic Efficacy and Tolerability of Molded Plastic Nasopharyngeal Swab (FinSwab) Compared to Flocked Nylon Swab in Detection of SARS-CoV-2 and Other Respiratory Viruses
Source: Microbiol Spectr. 2021 Oct 20;9(2):e00736-21. doi: 10.1128/Spectrum.00736-21 (PMC8528101; doi:10.1128/Spectrum.00736-21)

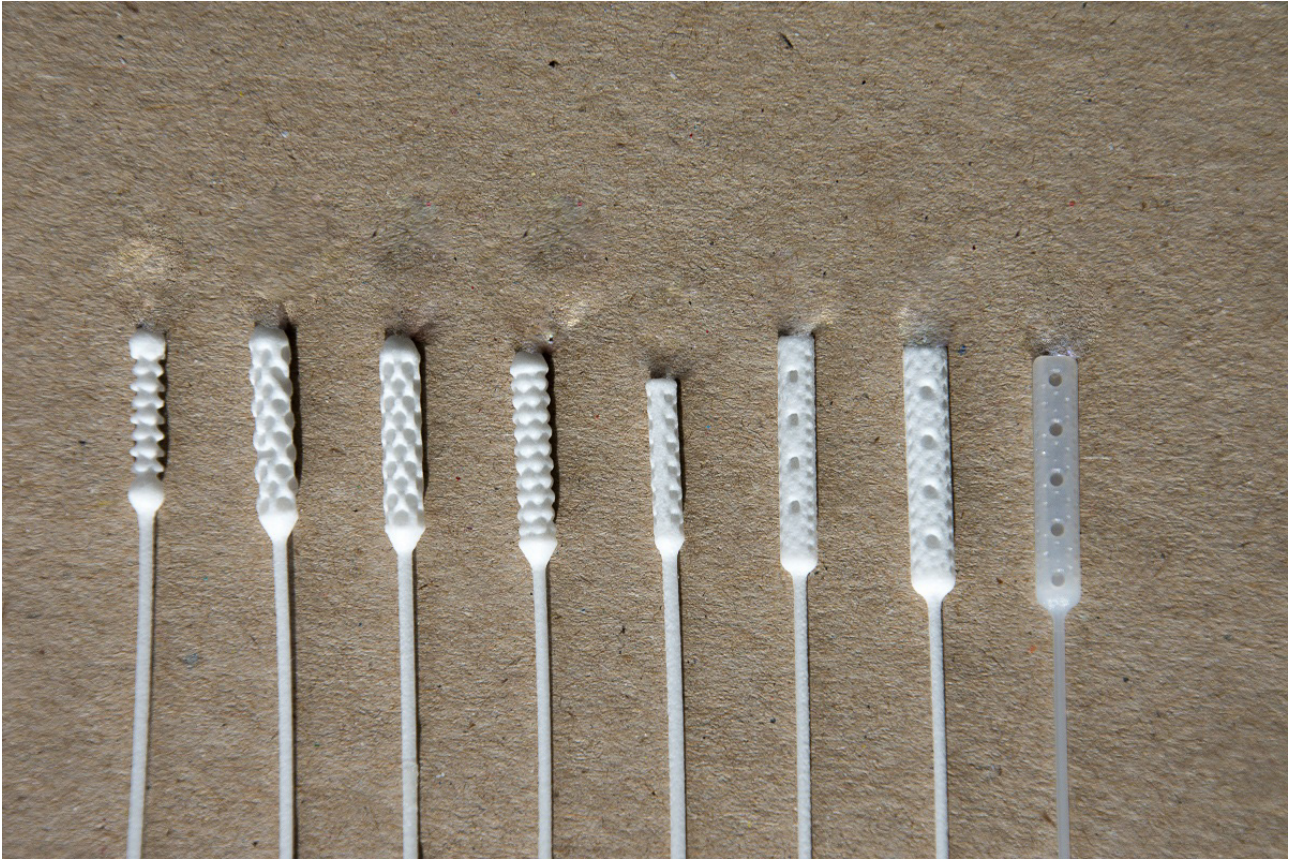

Figure S1. Swab prototypes and the final product (FinSwab; on the right).

Supplement: SUPPLEMENTAL FILE 1 — Supplemental material. Download SPECTRUM00736-21_Supp_1_seq2.pdf, PDF file, 0.6 MB [file spectrum00736-21_supp_1_seq2.pdf]
